# A Systematic Review of Heat Health Warning Systems: Enhancing the Framework Towards Effective Health Outcomes

**DOI:** 10.1007/s40572-025-00496-5

**Published:** 2025-08-21

**Authors:** Sai Venkata Sarath Chandra N, Jason Kai Wei Lee

**Affiliations:** 1 Present Address: School of Medicine and Dentistry, Griffith University, Gold Coast, Australia; 2 Heat Resilience and Performance Centre, Yong Loo Lin School of Medicine, National University of Singapore, Singapore, Singapore; 3 Human Potential Translational Research Programme, Yong Loo Lin School of Medicine, National University of Singapore, Singapore, Singapore; 4 Department of Physiology, Yong Loo Lin School of Medicine, National University of Singapore, Singapore, Singapore

**Keywords:** Heat waves, Personalized heat warnings, Heat alert threshold

## Abstract

**Purpose of Review:**

Heat Health Warning Systems (HHWS) reduce heat-related morbidity and mortality. We reviewed scientific studies on HHWS that use meteorological variables, local climate-epidemiological evidence, personalization, and built environment factors to determine heat stress thresholds. We identified key factors to enhance their precision and effectiveness.

**Recent Findings:**

We categorized the findings into three groups. First, most HHWS rely on temperature-mortality relationships. Second, future HHWS should integrate climate-epidemiology data, including cause-specific mortality and morbidity. Third, improvements can be made by incorporating local, built environment, and personalized factors. Our findings highlight a diverse range of factors that can influence the nature of heat warnings and contribute to improving HHWS.

**Summary:**

Temperature based HHWS are predominantly used across the world while other meteorological variables that include humidity and take actual health impact outcomes based on heat stress indices should be included for better protection. The precision of HHWS can be improved by tapping advancements in digital technologies to develop more targeted HHWS without the need for authorities to issue warnings, and by considering built environment, and personalized factors. The effectiveness of HHWS can be further improved by considering local climate-epidemiological evidence including morbidity and actual health outcomes.

## Introduction

With the world projected to heat by 2·4–3·5 °C by 2100, there is urgency to accelerate mitigation actions on greenhouse gas emissions and adaptation to prevent the devastating health and economic outcomes of a heating world [1]. Several parts of the world are already experiencing increased exposure to intense heat, more extreme weather and other calamities causing severe health risks due to climate change [2]. Some countries are most vulnerable to extreme heat due to socio-economic factors such as inadequate infrastructure, limited heat-health planning, and rapidly growing and ageing populations that strained the healthcare systems [3]. Even within countries, extreme heat poses significant dangers to certain population groups due to a combination of physiological, socio-economic, and environmental factors [4]. Individuals such as the elderly, those with chronic illnesses, and low-income communities are disproportionately affected as they live in areas with high exposure to heat, lack of access to adequate cooling, and have poor healthcare facilities [5]. Given the increasingly fatal nature of extreme heat on public health, many countries have taken steps to address its negative effects by developing and operationalizing Heat Health Warning Systems (HHWS) as one of the important protective strategies.

The HHWS are mechanisms aimed at minimizing negative health impacts of heat by alerting authorities and warning the public [6]. HHWS bring in significant benefits to public health by avoiding heat related mortality and morbidity [7]. For example, in Philadelphia, 117 lives were saved during 1995–1998 due to HHWS [8]. Introduction of a HHWS in France after the 2003 European heat wave resulted in saving an estimated 4400 lives during the 2006 European heat wave [9]. In Hong Kong, 5.5 and 6.4 deaths per day occurred due to ischemic heart disease and stroke when a HHWS was operationalized, compared to 6.1 deaths per day from ischemic heart disease and 6.8 deaths per day from stroke on days without a HHWS [10]. In South Korea, HHWS resulted in reduced mortality in the aged and socioeconomically vulnerable population [11]. However, though the evidence indicates that HHWS are effective in reducing mortality, there is a scope to improve HHWS by developing them for local conditions and increasing research on meteorological/climatological aspects of heat waves [12], and exploring other possibilities for their improvement [13].

Prior to identifying gaps in the functioning and improvement of HHWS, it is important to understand the basic working principles and procedures of HHWS. Ideally, HHWS are based on the establishment of thresholds of human health tolerance to heat and understanding determinants like index used for heat warnings, thresholds triggering warnings, rationale behind the threshold used, and identifying specific target groups [14]. The existing approach in many countries is based on identification of temperature thresholds using epidemiological studies that investigated historical temperature observations and mortality [15]. In this approach, HHWS are usually activated once temperature and/or other meteorological factors are forecast to breach predefined values expected to cause adverse health effects [16]. In some cases, the HHWS are implemented are based solely on regional weather observations and forecasts [17]. Such kind of HHWS do not take the temperature-epidemiological relationships to identification of thresholds for warnings.

Our study highlights the limitations of conventional HHWS that could be categorized either as - (i) partial absence of health factors (inclusion of temperature-mortality relationship without consideration of other health impacts like morbidity) or (ii) complete absence of health factors (solely based on historical temperature projections to identify a future condition as a heat wave) for identification of warning thresholds. This lack of systematic methodology for selecting a proper threshold poses a hindrance to authorities across various countries in establishing a HHWS [18]. To overcome this challenge, there are ongoing efforts to improve HHWS. There is also a debate to shift the nature of warnings by developing smart HHWS [19]. Smart HHWS are advanced, health-focused tools that combine weather forecasts with health data, vulnerability assessments, and community readiness to predict heat risks and guide targeted public health actions. Unlike conventional HHWS, which rely mainly on fixed meteorological thresholds to issue broad alerts, a smart HHWS focuses on health outcomes and adapt their warnings based on localized and individual risk factors [19] Table 1 presents an overview of conventional and smart HHWS discussing their features, data, and response strategies.

**Table 1:** Basic differences between conventional and smart HHWS

Feature	Conventional HHWS	Smart HHWS
Focus	Weather forecasts (e.g., high temperature warnings)	Public health outcomes (e.g., heat-related illness and mortality)
Data used	Primarily meteorological (temperature, humidity)	Integrated data: meteorological + health + social + individual vulnerability
Response	Generic alerts (all population)	Targeted, health-based interventions (e.g., activating health services, advising specific groups)
Scope	Often centralized and generic	Localized, risk- and population-specific
Adaptability	Static thresholds (e.g., > 40 °C triggers alert)	Dynamic thresholds based on risk (e.g., high humidity + urban poor = higher risk even at lower temperatures)

In this review, we identify the factors beyond meteorological variables that can influence heat warnings but are yet to be integrated into HHWS. Beyond examining the relationship between temperature and all-cause mortality to determine warning thresholds, considering the association between temperature and cause-specific mortality or morbidity due to specific health risks can also contribute to improvements in HHWS. For example, assessing temperature thresholds based on the onset of cardiovascular or respiratory illnesses can lead to effective health outcomes and strengthen heat action strategies [20]. Further, factors like urban planning, building, and housing aspects impact heat exposure of humans [21]. Personal heat exposure - the actual interaction between an individual and environmental conditions such as air temperature, radiant heat, humidity, and air velocity – is also an important factor as it can significantly elevate body core temperature and perceived discomfort, thereby exacerbating heat-related health impacts [22].

We identify the gaps in the functioning of existing HHWS across the world that do not yet focus on amplifiers of heat-health risk like personal and built environment parameters and the examined other factors that contribute to improvisations in HHWS. A recent systematic review by Kotharkar and Ghosh [23], examined ways to enhance heat-health action plans, including HHWS. In contrast, our study specifically focuses on improving HHWS and the determinants of heat warnings. We also examine the criteria for threshold determination and identified prospective parameters that can enhance the effectiveness of HHWS. Lastly, according to WHO/WMO report on Warning system development [24], HHWS vary across the geographical locations as extreme heat-health associations are specific to geography. Through our systematic review, we argue that HHWS must consider local climate-epidemiological factors and discuss the need for incorporation of built environment factors and personalized factors to improve precision in warnings. Technologically advanced countries can shift the focus towards personalized HHWS. Bridging the evidence gaps, we prescribe a model HHWS schematic building on the WHO/WMO HHWS schematic to improve health outcomes.

## Methods

### Search Strategy

For this review, we followed Preferred Reporting Items for Systematic reviews and Meta-Analyses (**PRISMA**) guidelines. We searched for studies in PubMed and Scopus from database inception to July 31, 2022. Key phrases used were “heat wave warning system”, “heat warning system”, “heat health warning system”, “hot weather warning system”, and “personalized heat warning” for each of the databases. We also conducted a secondary search in Google scholar for potential studies.

### Inclusion Criteria

We included studies focused on the working of HHWS, criteria for threshold determination, improvement in their functioning, and recent advancements in HHWS. We included studies that examined an association between HHWS and local and built environment factors. We included studies focused on monitoring individual physiological variables and personalized HHWS. We included peer reviewed research and review articles. We also included the WHO/WMO report ‘Heatwaves and Health: Guidance on Warning-System Development’.

### Exclusion Criteria

We excluded studies without a focus on HHWS. We also excluded non-English studies, gray literature which comprises technical documents, government reports, policy documents of countries, conference papers, and thesis studies.

**Fig. 1:**
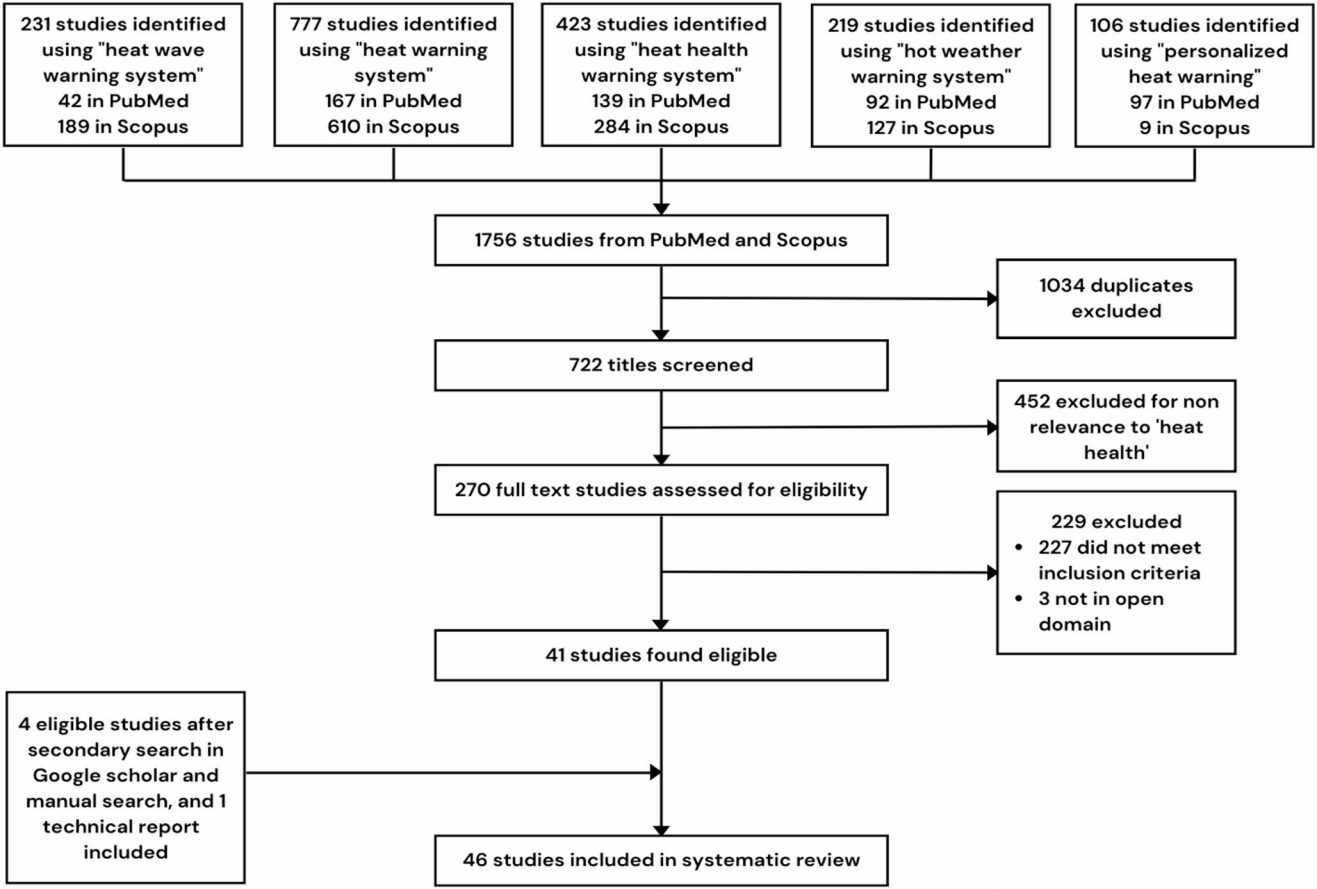
– PRISMA selection flowchart

## Results

We examined 46 studies which include 45 peer reviewed publications and one intergovernmental report using the search strategy (Fig. 1). These results are grouped into three categories. First category includes HHWS that comprise heat alerts, temperature thresholds, and variables. Second category includes studies that discuss how HHWS can be improved based on local climate-epidemiological evidence. The last category includes studies that discuss build environment factors, and the personalization of heat warnings (includes persons activity, clothing and age/sex/etc).

### The Nature of Existing HHWS and Variables Considered Across the World: Evidence from Scientific Literature

Two studies [10, 25] discussed HHWS in Asia. In Hong Kong, a Very Hot Weather Warning (VHWW) introduced in 2000 is based on the Weather Stress Index (WSI) [10]. WSI is calculated using net effective temperature (NET) which determines hot weather threshold in terms of ambient temperature, relative humidity, and wind speed [10]. In South Korea, HHWS is based on the threshold temperature which is the daily maximum temperature, and the value is determined by the relationship between excess mortality and temperatures [25]. These HHWS fit the context of partial absence of health parameters, highlighting the need for consideration of daily actual health impacts as morbidity or individual physiological conditions.

Nine studies [6, 26–33] discussed HHWS in Europe. In Germany, HHWS takes humidity, wind speed, solar and thermal radiation into account along with perceived temperature which measures thermal perception, and thermo physiological stress [6]. The German HHWS measures nocturnal indoor temperature of a modern standard building model which measures maximum of the average nocturnal temperatures in the west exposed and east exposed rooms [6]. In Switzerland, HHWS is based on heat stress indices relying on meteorological data [26]. A direct thermal index (hi) which combines temperature and relative humidity is used to determine the threshold factor [26]. In France, HHWS is based on the retrospective analysis of meteorological factors and mortality data [27]. In France, warning may be issued when the three-day averaged minimum and maximum forecasted temperatures have a probability of exceeding predefined local thresholds [28]. In Latvia, maximum daily temperatures are considered for two or more consecutive days and warnings are issued [29]. In London, HHWS thresholds are a daily maximum of 32 °C and a daily minimum of 18 °C [30]. In Portugal, HHWS is based on real time heat related mortality prediction rate calculated based on statistical analysis [31]. In Basque country of Spain, maximum and minimum temperatures for a duration of certain days are used to determine the heat wave 32. In Slovenia, warnings are based on the average and maximum daily temperatures [33]. The findings in Europe indicate that except for Germany which takes individual physiological factors into account, most HHWS fit the categorization of partial absence of health parameters without examining a relationship between meteorological variables and morbidity issues.

**Fig. 2:**
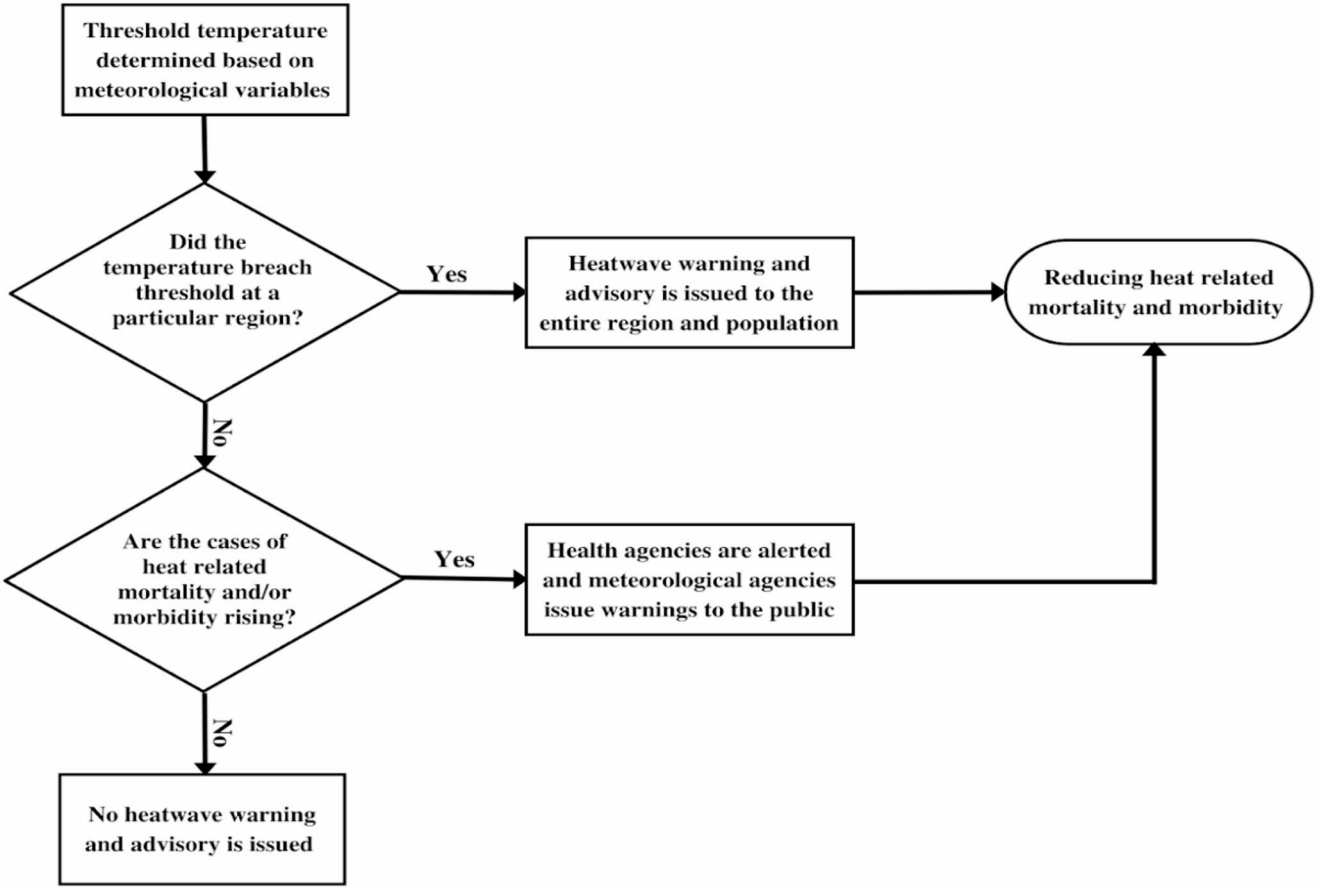
- A schematic of conventional HHWS, where warnings are based on meteorological variables—a common approach globally. The limitations of this model can be addressed through improvements illustrated in Fig. 5

Seven studies [34–40] discussed HHWS in North America. In Philadelphia, HHWS was introduced in the early 1990s based on a synoptic climatological procedure [34]. It was updated in 2003 with a real time web functionality and extended five days forecast [35]. HHWS in Dayton, Phoenix, and Toronto are also based on synoptic classification [35]. In Quebec (Canada), alerts are issued based on local Humidex thresholds [36]. In Ontario (Canada), a heat warning is triggered based on maximum and minimum temperatures for a duration of two or more days [37]. These threshold values for various zones in Ontario are determined by specific heat health evidence based on epidemiological analysis [37]. In Montreal and Greater Toronto Area (GTA), triggers for issuing warnings are based on spatial synoptic criteria, humidex threshold, minimum and maximum temperature thresholds [38]. In Canada, until 2015, heat alerts are based on the Canadian humidex without considering climatic variations and population responses [39]. However, the national heat alert program is being modernized by incorporating regional climatology, health evidence, heat event duration, and overnight temperatures [39]. In the USA, the National Weather Service (NWS) issues heat alerts based on the forecast of heat index which incorporates both temperature and relative humidity [40]. One study examined HHWS in Australia. In South Adelaide, an alert is issued if an average daily temperature (ADT) ≧ 32 °C for three consecutive days [41]. We observe a similar pattern to that of Europe in North America where meteorological variables determine the thresholds for heat warnings. Further, the findings from this section also indicate that most conventional HHWS lack targeted information and approaches for vulnerable at-risk populations. A simplified representation of how these systems operate is shown in Fig. 2

### Variables Proposed for Improving HHWS- Evidence on the Use of Other Meteorological Variables (Beyond Maximum Temperature) and Other Health Impact Variables (Beyond Mortality)

**Table 2:** The studies examined the association between heat exposure or heat effect variables and epidemiological evidence and proposed an optimal variable/combination of variables to improve HHWS

Authors	Country/region	Heat exposure or heat effect variables included and compared in the study	Epidemiological evidence considered in the study	Optimal variable or combination of variables identified for improvising HHWS
Cheng et al. 2019 [18]	Taiwan	Wet bulb globe temperature, temperature and apparent temperature	All-cause mortality, heat-related hospital admissions, and heat-related emergency visits	Wet bulb globe temperature
Lung et al. 2021 [42]	Taiwan	Wet bulb globe temperature and temperature	Heat-related emergency visits, heat-related hospital visits, and all-cause mortality	Wet bulb globe temperature
Tan et al. 2004 [43]	Shanghai	Arbitrary temperature threshold, Spatial synoptic for different character air mass	Mortality rate	Spatial synoptic air mass for moist tropical plus
Wu et al. 2020 [44]	Shanghai	One or the combination of maximum temperature, relative humidity, and duration	Morbidity and mortality data	Local variations to existing maximum temperature 35 °C as threshold
Lam et al. 2013 [45]	Hong Kong	Daily maximum and minimum temperatures, daily mean apparent temperature, daily net effective temperature, diurnal temperature range, and temperature change (mean temperature)	Mortality	Diurnal temperature range (DTR) and temperature change (mean temperature)
Li et al. 2014 [46]	China	Maximum temperature	All-cause mortality, Cardiovascular mortality, Respiratory mortality, Endocrine and metabolic mortality, Diabetes mortality, Digestive mortality, Genitourinary mortality	Maximum temperature at mortality due to all cause, cardiovascular, endocrine and metabolic, particularly diabetes
Chae et al. 2021 [25]	Seoul	Mortality based threshold, combined mortality and morbidity-based thresholds	Mortality and morbidity	Combined threshold temperature associated with mortality and morbidity
Kent et al. 2013 [47]	Alabama (USA)	Mean daily temperature, minimum daily temperature, maximum daily temperature, maximum daily apparent temperature, and maximum heat index	Preterm birth and non-accidental death	Relative mean temperature
Guo et al. 2012 [48]	USA	Temperature	Elderly mortality	Year by year temperature variability
Vaidyanathan et al. 2019 [49]	USA	Heat index at various levels including NWS	Hospitalizations	Heat index values lower than NWS
Metzger et al. 2010 [50]	New York (USA)	Heat index, temperatures, and spatial synoptic classification	Mortality	Maximum Heat Index
McElroy et al. 2020 [51]	San Diego	Maximum and minimum temperatures	Hospitalizations	Local geographical and epidemiological indicators
Issa et al. 2021 [52]	Canada	Maximum and minimum temperatures	All-cause mortality	Adjusted monthly thresholds of maximum and minimum temperatures
Vaneckova et al. 2011 [53]	Brisbane	Average temperature, maximum temperature, minimum temperature, apparent temperature, wet bulb globe temperature, humidex, thom discomfort index, and relative strain index	Mortality	Average temperature
Tong et al. 2014 [54]	Brisbane	Average temperature	Mortality and emergency hospital admissions	Average temperature
Faye et al. 2021 [55]	Bandafassi (Senegal)	A combination of maximum and minimum temperatures, apparent temperature at different percentiles and days as duration	Mortality	Minimum and maximum temperature threshold at 90th percentile within 3 consecutive days

Sixteen studies [18, 25, 42–55] identified effective meteorological variables for HHWS based on epidemiological evidence and emphasized the importance of using locally specific meteorological-epidemiological data. A study in Taiwan evaluated thresholds using three indicators— wet bulb globe temperature (WBGT), temperature, and apparent temperature—and three health outcomes: all-cause mortality, heat-related hospital admissions, and emergency visits [18]. Another study in Taiwan assessed WBGT and temperature thresholds in relation to heat-related emergency visits, hospital admissions, and all-cause mortality [42]. Both studies identify WBGT as an effective variable for HHWS development [18, 42]. In Shanghai, a synoptic air mass–based HHWS incorporating variables like dew point, cloud cover, and temperature was found more effective than using a fixed 35 °C temperature threshold [43]. A study in Shanghai evaluated five HHWS types—based on maximum temperature, relative humidity, or their combinations and duration—against heat-related morbidity and mortality data from local hospitals [44]. The findings suggest that threshold adjustments for maximum temperature and other variables are needed to tailor HHWS effectively for the Shanghai region [44].

A study in Hong Kong analyzed daily maximum and minimum temperatures, mean apparent temperature, net effective temperature, diurnal temperature range, and mean temperature change in relation to mortality data [45]. The study recommended incorporating diurnal temperature range and temperature change as key variables for an effective HHWS [45]. A study in China analyzed high temperatures and daily all-cause mortality in Harbin, Nanjing, Shenzhen, and Chongqing, highlighting the need for city-specific HHWS rather than a uniform national criterion [46]. In Seoul, a study revealed the limitations of fixed thresholds at 33 °C (Level 1) and 35 °C (Level 2), showing that significant heat-related morbidity and hospital admissions occurred even between 30 °C and 33 °C [25]. Combining threshold temperatures linked to both mortality and morbidity was found to enhance HHWS effectiveness [25].

In Alabama (USA), 16 heat wave definitions were evaluated to assess the relationship between heat exposure, preterm birth, and non-accidental death [47]. The 16 heat wave definitions used various heat exposure metrics, including mean, minimum, and maximum daily temperatures, maximum apparent temperature, and heat index [47]. The study also found that relying solely on absolute thresholds in the NWS alert system may be suboptimal for protecting public health [47]. A study investigated the link between high temperatures and elderly mortality in the USA [48]. The findings showed that in some years, high temperatures correlated with increased mortality, while in others, they were linked to decreased mortality [48]. This underscores the need for HHWS to account for year-to-year risk variations rather than relying on multi-year average risk [48]. A study in the USA found that heat-related hospitalizations in some regions begin at heat index levels below the NWS alert thresholds [49].

A study assessed heat index, maximum, minimum, and average temperatures, along with spatial synoptic classification (SSC), as predictors of natural-cause mortality in New York City during summer months from 1997 to 2006 [50]. The study found that a maximum heat index of 95–100 °F or higher was linked to increased mortality over the following three days [50]. In San Diego, a study found regional variation in temperature thresholds for hospitalizations, suggesting HHWS accuracy can be improved by incorporating local epidemiological, geographic, and climate-specific data [51]. A Canadian study recommended monthly adjustments to mortality-related temperature thresholds rather than using fixed thresholds for the entire summer [52]. The study found that maximum and minimum temperature pairs varied monthly in relation to excess mortality, highlighting the need for a data-driven, adaptable HHWS that accounts for human acclimatization and seasonal climate variability [52].

A study in Brisbane examined meteorological variables included average temperature, maximum temperature, minimum temperature, apparent temperature, wet bulb globe temperature, humidex, thermal discomfort index, and relative strain index [53]. The variables were evaluated against mortality as epidemiological data [53]. The findings indicate that average temperature performed comparably to biometeorological indicators and can serve as an effective variable [53]. Using average temperature as a metric, another study in Brisbane examined mortality and emergency hospital admissions [54]. The study found increased risks of mortality and emergency admissions when average temperatures exceeded the 90th, 95th, or 98th percentile for two, three, or four consecutive days, respectively [54]. The study proposed a three-tiered HHWS based on the average temperature [54]. A study in Senegal found that using both minimum and maximum temperature thresholds at the 90th percentile over three consecutive days was most effective for developing a HHWS [55]. A summary of these studies is presented in Table 2.

### Studies Focusing on Targeted Heat Warnings Based on Built Environment and Personalized Factors

Two studies [17, 56] examined the value of built environment factors to develop precise warnings for individual households based on indoor temperatures. One study proposed a high resolution indoor HHWS based on indoor temperature thresholds using a Cumulative Heat Index [17]. Another study proposed a generic residential building model that estimated the minimum, average, and maximum room temperatures for an effective residential HHWS [56]. Our evidence indicates that except for Germany, we do not find any HHWS considering built environment variables like indoor temperature in the determination of heat warning thresholds. We also find a significant research gap with only two studies examining the relationship between heat warnings and built environment factors.

Six studies [57–62] discussed personalization of warnings based on advancements in technology and experimental personalized HHWS. One study designed a prototype device to monitor changes in skin temperature for assessing body core temperature, blood oxygen saturation, heart rate, and galvanic skin response to determine heat stress effects on individuals [57]. The prototype device was found to be effective in determination of personal heat risk factors [57]. Four studies [58–61] examined the working of projects Heat Shield, ClimApp, and Worklimate on personalized heat warnings that use wearable, mobile application, and GPS technology to create more precise protection for individuals. One study demonstrated the effectiveness of automated phone warnings [62]. It was found that an automated phone warning system improved heat adaptation, reduced the use of health services by vulnerable groups, and was effective on vulnerable populations [62]. Overall, the studies on personalization of heat warnings are also significantly lower compared to those examining the relationship between heat warnings and health evidence as mortality or morbidity.

**Fig. 3:**
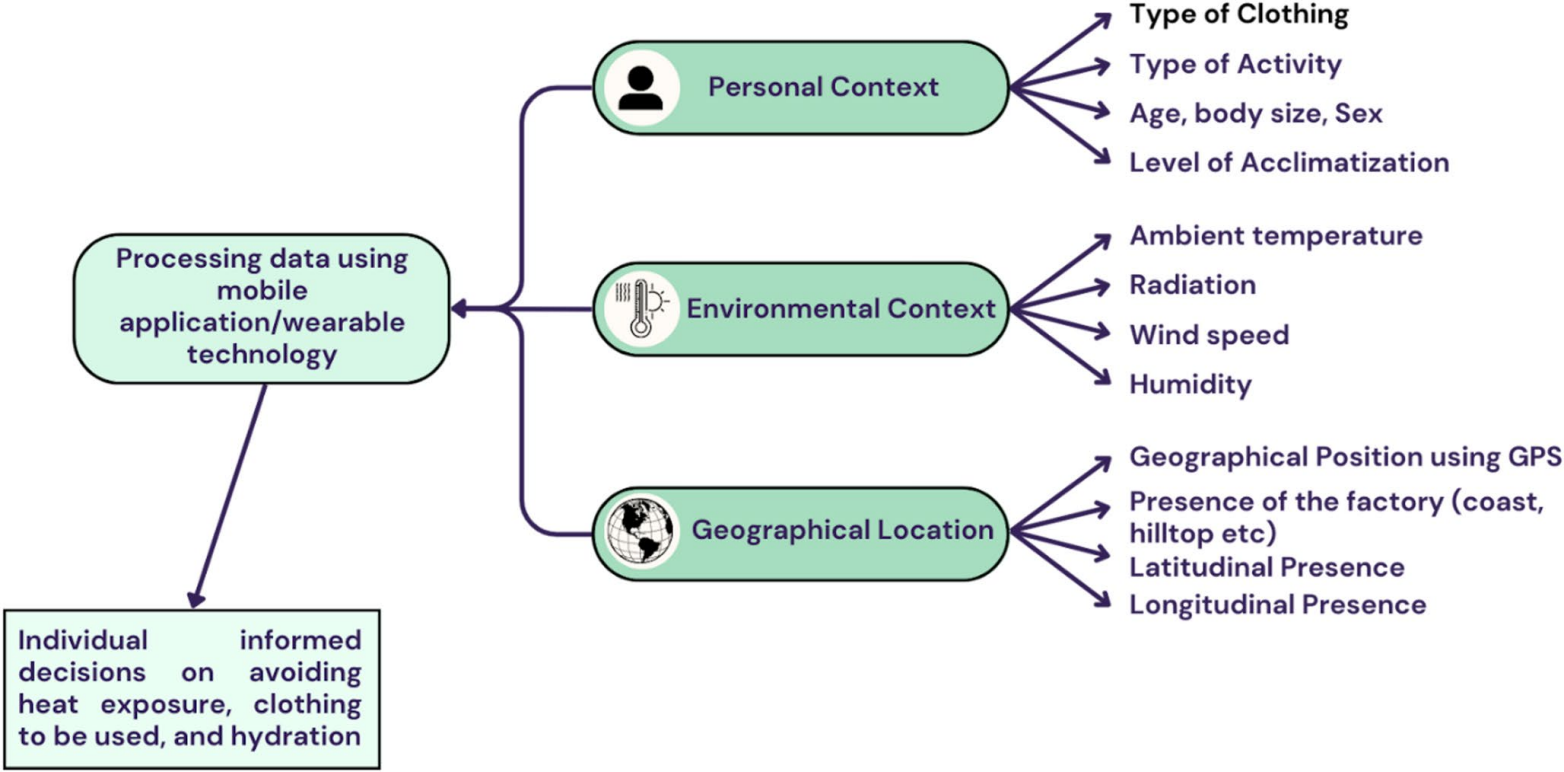
- A schematic representation of the working of personalized HHWS

The evidence from [58–61] indicates that personalized HHWS contain information that targets specific at-risk populations. For example, ClimApp offers profile-based outputs for individual users, as well as caregivers of children and the elderly, delivering tailored recommendations to protect these at-risk groups [59]. Project Heat Shield and Worklimate also offer targeted warnings, specifically addressing the needs of vulnerable groups such as outdoor workers exposed to extreme heat [58, 61]. These personalized HHWS capture individual-level variation by considering differences in vulnerability, exposure, and heat response capacity. They integrate personal, occupational, and environmental data to deliver tailored guidance. Key physiological factors—such as age, fitness, body composition, underlying health conditions, and heat acclimatization—are also included, as they significantly influence heat tolerance. A simplified schematic illustrating the functioning of personalized HHWS is presented in Fig. 3.

**Fig. 4:**
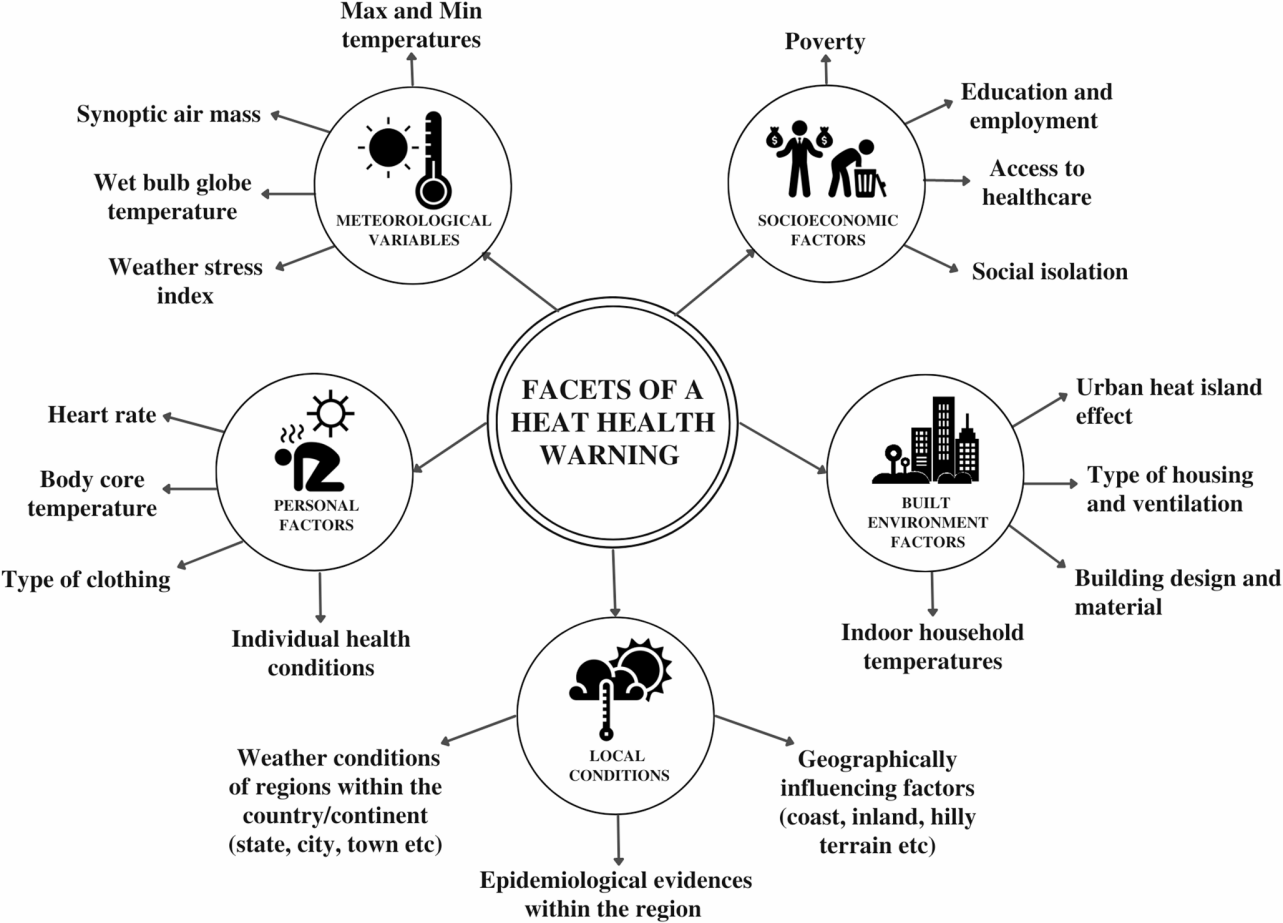
- General inputs that have the potential to influence heat warnings or the effectiveness of HHWS. While meteorological variables are popular inputs for HHWS, we highlight the need for designing HHWS based on the variables as indicated

Overall, the summary of findings indicates a diverse range of factors that can influence heat warnings (Fig. 4). Most HHWS across the regions of the world also rely on meteorological variables in determining the warning thresholds. They are either based on temperature-mortality relationship or historical projections without any health impact consideration. Studies to improve the effectiveness of HHWS are also dominated by the ones discussing meteorological variables and epidemiological evidence linkage. Across the countries, predominantly used meteorological variables in HHWS include daily maximum temperature, relative humidity, and a combination of daily maximum and minimum temperatures. Research in some regions highlight the importance of considering WBGT as a variable. Widely used epidemiological evidence for consideration in future HHWS include all-cause mortality, heat related morbidity, and heat related hospitalizations. A combination of the above variables is used by various countries/continents in determining the warning threshold for a HHWS and the alerts are disseminated in a top-down manner without any specific focus or actionable information that target at-risk groups. Further, the results also indicate an emerging trend towards personalized HHWS that contain tailored strategies specific to at-risk groups. In the discussion section, we highlight the working of HHWS by examining the pros and cons of conventional and personalized HHWS and then present possible improvements to the overall working of HHWS.

## Discussion

Though there exists literature examining effective meteorological variables for HHWS to reduce heat related mortality and morbidity, there is a need for more research in the future to identify an effective variable or a combination of variables based on meteorological-epidemiological evidence across the countries that can improve the effectiveness of HHWS. Further, given the geographical diversity, a uniform meteorological-epidemiological criteria may not be useful to determine a heat alert threshold and there cannot be a one size fits all approach while determining a heat alert threshold of a HHWS. There is a need to focus on local weather and climatic conditions. To explain further, entire country with a large geographical stretch and variations in climatic conditions cannot rely on mean maximum and minimum temperature, wet bulb globe temperature, or on spatial synoptic air mass for HHWS. Inferring from the findings in San Diego [51], we further make a case for localization of heat warnings within the country to province/state level, city level, and even town levels.

We also focus on the built environment factors that influence how people experience heat as evident in the studies [17, 56]. The amount of indoor heat exposure impacts health. The type of housing and ventilation influences indoor heat exposure. People in such households need a special focus while disseminating heat warnings. However, to overcome the challenge of identification of such households susceptible to extreme indoor heat, we emphasize on measuring indoor temperatures by setting up heat monitoring devices within individual households, thus resulting in the development of indoor household HHWS that can quantify risk exposure at the household level.

We then shift the focus towards personalization of heat warnings without the need to rely solely on lead agency to issue extreme heat warnings as discussed in the studies [57–62]. There are certain physiological parameters sensitive to heat that can determine the effect of heat stress on individuals. The progress in digital and wearable technology has made monitoring changes in these parameters easier. In the light of these advancements, wearable technologies can be combined with mobile application and geospatial technologies for the development of personalized HHWS. Heat warnings can consider additional factors like an individual’s physical activity, type of clothing, and body characteristics along with meteorological forecasts based on GPS. Two personalized HHWS, ClimApp and Worklimate can serve as models for the future development of personalized HHWS. We focus on improving the precision of HHWS targeting population groups vulnerable due to socioeconomic conditions, age, occupation, and preexisting health conditions. These at-risk groups need alerts when temperatures breach a certain threshold that must usually be lower than normal warning threshold temperatures. Such targeted action strategies by issuing heat warnings to these groups can result in likely benefits as reduced mortality and morbidity.

This review also discusses a shift in heat warnings to a human centric level that can occur using digital technology [58–61]. Developing mobile applications that act as bridges to connect external weather conditions and internal human physiological conditions can be useful. First, the digital interface takes inputs from the external environment (outside temperatures, weather and climatic conditions) and warns individuals about heat. Second, the digital interface also monitors changes in human physiological parameters and warns individuals about possible illness. Third, we combine both the components where the digital interface can simultaneously monitor physiological changes in relation to external temperature changes. We also suggest developing automated phone warnings like the Telephone Sante project tested to be effective in Longueuil of Montreal, Canada complementing HHWS [62].

Findings suggest that conventional HHWS rely on fixed thresholds to issue broad public alerts. Their strengths lie in simplicity, low cost, and ease of implementation, making them suitable for wide outreach. However, they often fail to protect vulnerable populations, as they do not account for individual-level risk factors such as age, health status, or socioeconomic conditions. As a result, these systems may not trigger timely or targeted protective actions. In contrast, personalized HHWS integrate meteorological, health, and social data to dynamically assess risk and issue tailored advisories. They can identify high-risk individuals and enable context-specific responses, offering greater effectiveness in reducing heat-related morbidity and mortality. However, their implementation is resource-intensive and requires technological infrastructure and data integration. To bridge the gap between these two approaches, community engagement plays a critical role. Strengthening community-level resilience and involving key local stakeholders can enhance the reach, relevance, and effectiveness of heat warnings—particularly in resource-constrained settings [63].

**Fig. 5:**
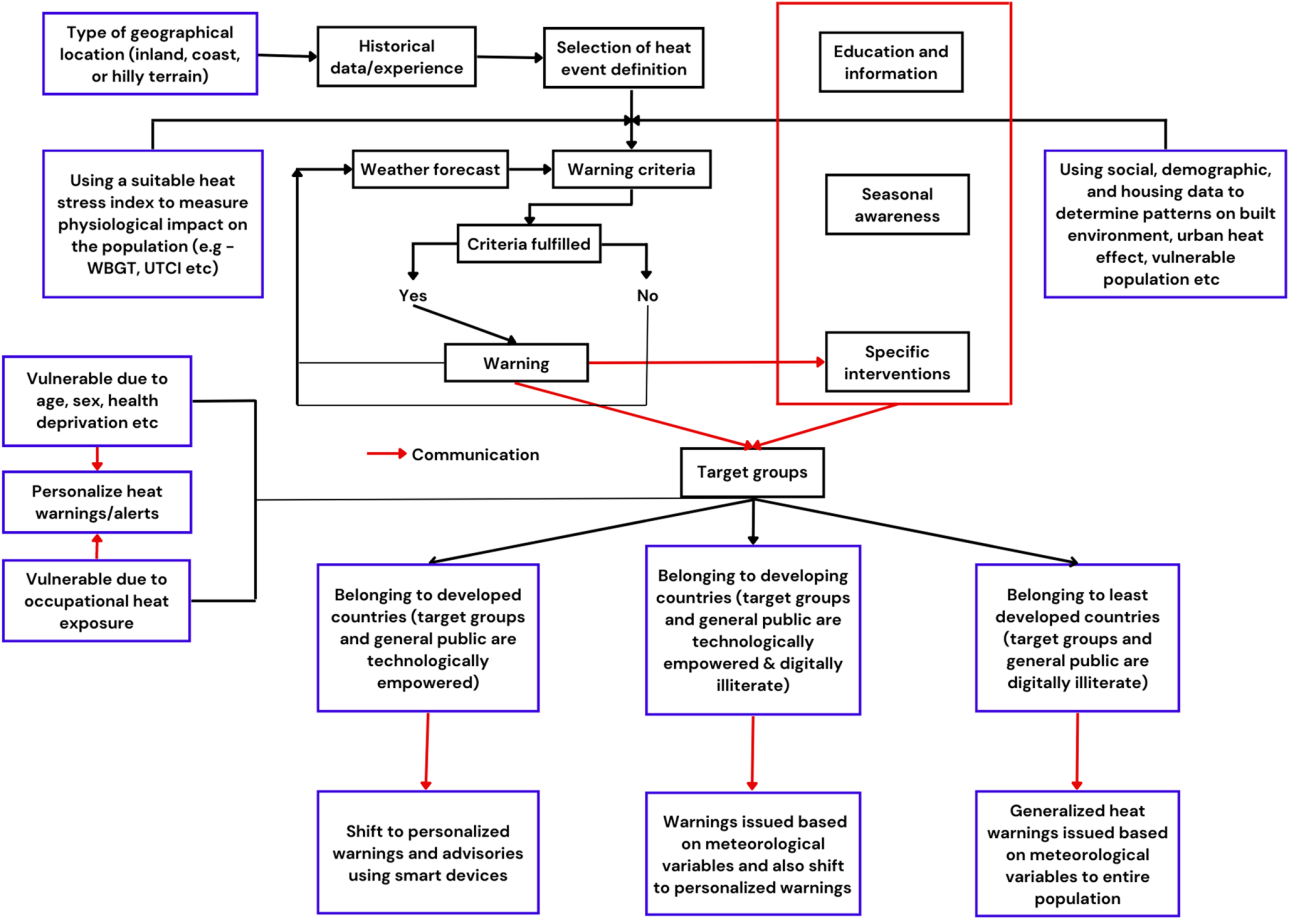
- HHWS schematic developed from the model HHWS of WHO/WMO [24] (boxes in blue color represent the newly enhanced framework). Based on the gaps identified in the findings, we propose additional criteria that include consideration of nature of location, use of a heat stress index, and the use of technology for targeted heat warnings

We examine published guidance on HHWS by WHO/WMO [24] and identify gaps where HHWS can be developed towards more specialized systems considering the geographical location, population vulnerability, and examining the physiological impact on the population at a larger level using a suitable ‘heat stress index’. Our analysis attempts to bridge these gaps by proposing a new schematic of HHWS that can be used for development in the future by considering a set of variables as indicated in our findings and shifting the focus towards personalization wherever feasible (Fig. 5). However, we acknowledge that our case for personalization of heat warnings is practical only in countries where technological advancements are higher, people are digitally empowered, and there is proliferation of smart devices. In countries that are less technologically advanced, and people are not well accustomed to using smart devices, personalization of warnings may not have practical applicability. In such less technologically advanced countries, we recommend conventional HHWS as indicated in Fig. 2 along with corresponding heat health advisories to targeted vulnerable high-risk groups and general advisories to non-vulnerable groups. Conventional HHWS must continue to be implemented globally, with necessary enhancements—such as incorporating heat stress indices and shifting from all-cause to cause-specific mortality and morbidity as health-impact evidence.

While strengthening HHWS is essential, it is equally important to address the issue of heat warning fatigue. This refers to a phenomenon where individuals become desensitized to alerts, causing them to ignore or underestimate the warnings. Key contributors include frequent or repetitive warnings [18], the perception of heat as normal [64], limited risk awareness [65], socioeconomic constraints [66], and ineffective communication [67]. Our study suggests that warning fatigue can be addressed through key improvements to HHWS: (1) using trusted public platforms and clear, simple messaging in conventional systems; (2) enhancing precision and targeting through personalized HHWS; and (3) improving geographic specificity by incorporating local factors. As HHWS research evolves, there is a growing need to better understand and address heat warning fatigue.

The key idea of our study is to propose a reformed HHWS schematic (Fig. 5). Unlike the previous studies on HHWS that reviewed meteorological variables, we provide an analysis for improving HHWS by taking local meteorological-epidemiological evidence, built environment, and personalized factors into account. However, several limitations of this study should also be noted. First, while HHWS based on meteorological variables are practically in use across the world, personalized HHWS and hypotheses for the inclusion of built environment factors are at the experimentation stage. Second, though heat warnings issued by agencies rely on time as series of consecutive days above a threshold, we did not find any studies that discuss how time plays a role in enhancing the effectiveness of heat warnings. Third, there is not enough evidence on the efficacy of household and personalized HHWS in reducing mortality and morbidity.

## Conclusion

To summarize, there is a need for upgradation of HHWS currently in use in most of the countries. We do not argue that climate-based predictive conventional HHWS are ineffective; rather, we aim to identify areas for improvement to strengthen their relevance in a warming world. HHWS rely on meteorological data, and temperature alone is often used, but by including humidity levels and actual health impacts based on certain heat stress indices (WBGT or Heat Index) can make the HHWS more effective. This means that meteorological services should adopt “Heat Stress Index” that incorporate humidity, wind speed, and solar radiation along with temperature in their HHWS. This would move HHWS beyond the widespread reliance on maximum temperature alone for extreme heat warnings.

HHWS should include built environment adjustment estimates like consideration of indoor temperatures to highlight heat risks in vulnerable areas at a further local level. We also argue that there is a need to personalize heat warnings wherever they are practical. Technologically advanced countries can shift the focus of heat warnings to enhance precision by doing away with the need for agencies to issue warnings and by promoting mobile applications and digital tools. In the future, as a first step, we recommend developing conventional HHWS in all the countries suffering adverse consequences of heat yet do not have HHWS. Necessary reforms to warning mechanisms can be made further as highlighted by this study. Considering global heating, we conclude by making a case for more countries to develop and upgrade HHWS with innovative mechanisms to integrate the personal, local climate-epidemiological evidence, and built environment factors.

## Key References

- Casanueva A, Burgstall A, Kotlarski S, Messeri A, Morabito M, Flouris AD, et al. Overview of Existing Heat-Health Warning Systems in Europe. Int J Environ Res Public Health. 2019 Aug;16(15):2657. 10.3390/ijerph16152657.
-
  - This study discussed the working of Heat-Health Warning Systems in Europe.
- McGregor GR, Bessemoulin P, WMO. Heatwaves and health guidance on warning-system development. Genf: World Meteorological Organization; 2015. https://www.who.int/publications/m/item/heatwaves-and-health--guidance-on-warning-system-development.
-
  - This work presents a guidance on the development of Heat-Health Warning Systems for countries across the world.
- Eggeling J, Rydenfält C, Kingma B, Toftum J, Gao C. The usability of ClimApp: A personalized thermal stress warning tool. Clim Serv. 2022 Aug 1;27:100310. 10.1016/j.cliser.2022.100310.
-
  - This study discussed how a personalized Heat-Health Warning System can be developed and can work.
- Kotharkar R, Ghosh A. Progress in extreme heat management and warning systems: A systematic review of heat-health action plans (1995–2020). Sustain Cities Soc. 2021 Oct 1;76:103487. 10.1016/j.scs.2021.103487.
-
  - This study provided an overall progress and overview on the development of heat warning systems across the world.

## Data Availability

No datasets were generated or analysed during the current study.
